# Synthesis of Marine Natural Products and Molecules Inspired by Marine Substances

**DOI:** 10.3390/md19040208

**Published:** 2021-04-08

**Authors:** Emiliano Manzo

**Affiliations:** Bio-Organic Chemistry Unit, Institute of Biomolecular Chemistry of the National Research Council (CNR), Via Campi Flegrei 34, 80078 Naples, Italy; emanzo@icb.cnr.it

The sea covers more than 70% of Earth’s surface and contains more than 300,000 organisms with huge biodiversity. These organisms represent an enormous tank of substances whose chemical structures are the result of the enormous ecological pressure for survival. Given their high chemical diversity, biological activity, biochemical specificity and other crucial molecular properties, marine natural products have always played a crucial role in the search for and discovery of novel bioactive molecules potentially useful for pharmacological applications. In fact the development of new “lead” compounds from the sea has always been—and is one of the main purposes of drug discovery [1].

The study and advanced progress of these products cannot be separated from the development of chemical synthesis and synthetic strategies aimed at the preparation and optimization of these substances and/or analogs, opening the way to new classes of biologically active compounds with pharmacological potential.

This Special Issue, comprising eight articles and one review, describes the synthetic methodologies and biological activity of different classes of bioactive marine metabolites and analogs crucial to favor pharmacological applications of these molecules.

Esposito et al. [2], with the aim to optimize the antimicrobial activity of marine derived imminosugars, efficient glycomimetics, reported a synthetic strategy for the preparation of their lipophilic analogs characterized by promising antimicrobial activity due to improved internalization within the bacterial cell. This behavior was in fact favored by major lipophilicity of these molecules. The one-pot strategy involved the conjugation of iminosugars with lipophilic moieties, such as cholesterol, through a cleavable succinic acid linker that would positively contribute to substance transfer and release within the bacterial cell. The synthetic procedure, favored by combined use of the polymer-supported triphenylphosphine, molecular iodine and imidazole [3] in a one-pot strategy with cholesterol, succinic acid and unprotected iminosugars, led to the final compounds in high yields.

Mazzotta et al. developed a synthetic strategy for the preparation of different new marine-derived labdane diterpenes analogs, characterized by the homodrimane backbone bearing flexible tails with various chemical properties [4]. They start from the commercial (+)-sclareolide. In particular, different modifications, such as amide, ester and carboxylic acid functions, were introduced on the sclaerolide. The subsequent derivatization reaction led to a series of homodrimane analogs characterized by important biological activities involving the activation of the transient receptor potential channel subfamily V member 4 and 1 (TRPV4 and TRPV1) channels [5,6,7] that have recently emerged as a pharmacological target for several respiratory diseases, including the severe acute respiratory syndrome coronavirus 2 (SARS-CoV-2) infection. Chemical determinants crucial for TRPV4 and TRPV1 antagonism were identified by structure–activity relationships. This study represents the first report of semisynthetic homodrimane TRPV4 antagonists, selective over TRPV1, and potentially useful as pharmacological tools for the development of novel TRPV4 channel modulators.

Martinez et al. showed a concise strategy for the preparation of sponge derived tetracyclic meroterpenoids characterized by a sesquiterpene skeleton linked to a phenolic or quinone part [8]. Aureol, strongylin A, cyclosmenospongine and smenoqualone are representative examples of this class of bioactive compounds. The preparation of aureol was made possible by using a short and efficient synthetic route relying on a C–C bond-forming reaction between albicanal and an aryllithium-derivative and a sequence of 1,2-hydride and 1,2-methyl shifts mediated by BF_3_•Et_2_O as activator and water as initiator. Aureol and 5-epi-aureol obtained by this strategy are key intermediates opening the way for the synthesis of a large number of natural and synthetic tetracyclic meroterpenoids with potential antitumor and antiviral activity.

Ouyang et al. described the first eight steps of the total synthesis of 5′-*O*-α-D-Glucopyranosyl Tubercidin, a disaccharide 7-deazapurine nucleoside characterized by fungicidal activity [9]. The chemical approach, based on trichloroacetimidate strategy, consisted in one-pot Vorbrüggen glycosylation of protected ribose with 6-chloro-7-bromo-7-deazapurine and stereoselective α-*O*-glycosylation of 7-deazapurine nucleoside derivative with 2,3,4,6-tetra-O-benzyl-glucopyranosyl trichloroacetimidate.

Zhang et al. showed how the substitution on the defined position on the chemical structure of bioactive molecules analogs could be a determinant for biological activity [10]. In particular, the authors experienced that fluorination at the C18 position of the marine-derived largazole showed good tolerance towards inhibitory activity and selectivity of histone deacetylases (HDACs). Further substitution on valine residue in the fluoro-largazole’s macrocyclic moiety with S-Me l-Cysteine or Glycine moieties was performed, leading to derivatives with totally different biological activity. In particular the S-Me l-Cysteine-modified analog displayed enhanced inhibition of all the tested HDACs. Furthermore, a molecular modeling analysis provided a rational explanation and structural evidence for the enhanced inhibitory activity. This new finding will aid the design of novel, potent HDAC inhibitors.

Eldehna et al. designed and synthetized different anticancer marine inspired indoles and bis-indoles, as Topsentin and Nortopsentin analogs [11]. This synthesis was based on replacing the heterocyclic spacer in the natural leads with a more flexible hydrazide linker between indole rings. In this approach, in fact, the rigid heterocyclic spacer in the marine natural products Topsentin and Nortopsentin was replaced by the flexible linker and this change resulted in the development of bis-indole scaffold with promising in vitro antitumor activity toward breast cancer cell lines. Different analogs were prepared and all the synthetic bis-indoles were characterized for antiproliferative action against human breast cancer (MCF-7 and MDA-MB-231) cell lines. These results suggested that the reported bis-indoles are good lead compounds for further optimization and development as potential efficient anti breast cancer drugs.

Pemha et al., starting from recinoleic acid, synthetized a series of 1-*O*-alkylglycerols containing different functional groups (methoxy, amide, fluoro, azide and hydroxy) on the alkyl chain and antimicrobial activity was evaluated for all compounds [12]. The hydroxy derivative displayed more promising and significant activity. It was evident that synthetic non-natural 1-*O*-alkylglycerols can be further explored as a new source of drugs.

Imperatore et al. reported the chemical synthesis of two prenyl-quinones and their corresponding dioxothiazine fused quinones [13]. The synthesis of the prenylated compounds was performed through an efficient and versatile strategy designed and developed in order to easily reproduce the chemodiversity within the thiazinoquinones library. These molecules were inspired to an antitumor marine compound, a geranylquinone with 1,1-dioxo-1,4-thiazine ring isolated from the ascidian *Aplidium conicum* and named aplidinone A [14,15,16]. The synthetic molecules showed a comparable toxicity against three different human cancer cell lines, breast adenocarcinoma (MCF-7), pancreas adenocarcinoma (Bx-PC3) and bone osteosarcoma (MG-63).

Vessella et al. in their review have collected different and representative reports regarding the total synthesis of fucosylated chondroitin sulfate (fCS) oligosaccharides and semisynthetic strategies to obtain fCS oligosaccharides and low molecular weight polysaccharides [17]. The importance of this review was based on the various potential biomedical applications of marine derived fCS, among them its anticoagulant activity. This is the first report on (semi)-syntheses of (macro)molecules resembling the structure of natural fCS polysaccharides. Total synthetic and/or semi-synthetic strategies have been discussed, underlining the advantages and drawbacks for each approach and also reporting the main results on the structure–bioactivity relationships. The authors are sure that the research targeting the (semi)-synthesis of fCS oligo- and polysaccharides and analogs will attract a growing interest in the next few years.

As guest editor, I am grateful to all the authors who contributed their excellent results to this Special Issue, all the reviewers who carefully evaluated the submitted manuscripts, and *Marine Drugs* for their support and kind help.
